# The Emerging Burden of Opioid Poisoning in Brazil, 2019–2025: A Nationwide Epidemiological and Toxicovigilance Analysis

**DOI:** 10.3390/ph19070994

**Published:** 2026-06-26

**Authors:** Luíza Siqueira Lima, Diancarlos Pereira de Andrade, Viviane Serra Melanda, Ana Tereza Bittencourt Guimarães, Cláudia Sirlene Oliveira

**Affiliations:** 1 Instituto de Pesquisa Pelé Pequeno Príncipe, Curitiba 80230-020, PR, Brazil; luizaelima04@gmail.com (L.S.L.); diancarlospa@gmail.com (D.P.d.A.); ana.guimaraes@professor.fpp.edu.br (A.T.B.G.); 2 Faculdades Pequeno Príncipe, Curitiba 80250-060, PR, Brazil; 3 Secretaria de Estado da Saúde do Paraná, Curitiba 80230-140, PR, Brazil; viserramel@gmail.com

**Keywords:** opioids, opioid-notified poisoning, exogenous intoxication, toxicovigilance, Brazil, epidemiology, public health surveillance

## Abstract

**Background:** Despite increasing opioid use in Brazil, the national epidemiological profile of opioid-related poisonings remains insufficiently characterized. **Objective:** To characterize opioid-notified poisonings reported in the Sistema de Informações sobre Agravos de Notificação (SINAN—Notifiable Diseases Information System), accessed through Departamento de Informática do Sistema Único de Saúde (DATASUS—Department of Informatics of the Unified Health System), between 2019 and 2025. **Methods:** This retrospective descriptive study used secondary national surveillance data from publicly accessible databases. Records of exogenous poisonings related to medications and drugs of abuse were screened, and notifications involving opioid analgesics were identified and standardized. Descriptive analyses were performed for demographic, clinical, and exposure-related variables. Bivariate analyses and multivariable logistic regression, regression models were conducted for selected outcomes. Incidence rates were estimated by year and federative unit, and temporal trends were assessed using generalized linear mixed models. **Results:** Between 2019 and 2025, 1,127,265 poisonings related to medications and drugs of abuse were reported in Brazil, of which 12,645 involved opioids. The opioids most frequently implicated in notifications were codeine (38.65%), tramadol (33.98%), and morphine (17.86%). Most cases occurred in women (70.3%), in individuals aged 26–50 years (47.8%), and in residences (85.6%). Digestive exposure predominated (92.3%), and suicide attempt was the main circumstance (73.5%). Most patients recovered without sequelae (75.1%), whereas 1.6% died due to exogenous intoxication. Co-exposure information was classifiable in 9573 records, most commonly involving opioids and medications. In multivariable analyses, suicide attempts were associated with female sex (aOR = 1.98; 95% CI: 1.68–2.34), residence-based exposure (aOR = 8.95; 95% CI: 6.29–12.72), and co-exposure (aOR = 2.17; 95% CI: 1.82–2.60). Hospitalization was less likely among females (aOR = 0.83; 95% CI: 0.75–0.91) and more likely with co-exposure (aOR = 1.14; 95% CI: 1.02–1.27). Serious outcomes were associated with older age (aOR = 1.017; 95% CI: 1.009–1.026), while residence-based exposure and suicide attempt showed lower odds. A significant increasing temporal trend was identified, with higher reported notification rates observed in the South and Southeast regions. **Discussion:** The predominance of suicide attempts and residential digestive exposures suggests that the notification profile captured by SINAN/DATASUS is predominantly shaped by intentional self-poisoning and household medication availability, while still representing a broader toxicovigilance scenario involving abuse, habitual use, adverse reactions, and other exposure contexts. The contrast between the most frequent notification profile and the profile associated with serious outcomes indicates that occurrence and severity may follow different epidemiological patterns. Therefore, these findings should be interpreted as a toxicovigilance signal reflecting multiple exposure contexts rather than as evidence of a single opioid-use pattern. **Conclusions:** Reported opioid-notified poisonings in Brazil increased over the study period and were predominantly associated with domestic exposure, suicide attempts, and co-exposure to other substances. These findings highlight the clinical and public health relevance of opioid-notified poisonings and support the need for strengthened surveillance, improved reporting quality, and preventive strategies addressing both opioid use and mental health. **Limitations:** Underreporting, missing data, regional reporting differences, and possible misclassification in SINAN/DATASUS records; therefore, associations, temporal increases, and projections should be interpreted as exploratory, and hypothesis generating.

## 1. Introduction

Although opioids are essential medicines for pain management, opioid-related poisonings may arise from multiple contexts, including therapeutic use, medication errors, intentional self-poisoning, abuse, habitual use, and polysubstance exposure [1]. Globally, in countries such as the United States and Canada, what is known as the “opioid crisis” has emerged, where increased opioid prescribing, non-medical use, and overdose deaths have made opioid-related harms a major public health concern [1,2]. Worldwide, the availability of opioids varies according to public policies regulating their control and access, the availability of analgesic treatments, and the local epidemiological profile of the most prevalent diseases requiring pain management [3,4,5].

Of the approximately 600,000 drug-related deaths recorded worldwide in 2019, around 80% were linked to opioid use, with roughly 25% of these (approximately 125,000 deaths) resulting from overdose. In the United States, 70,630 opioid overdose deaths were reported in 2019, with at least half involving synthetic opioids. Although opioid-related fatalities are high, non-fatal overdoses are even more prevalent and occur more frequently than fatal events [6]. Globally, an estimated 296 million people (5.8% of the population aged 15–64 years) reported using drugs at least once in 2021, including approximately 60 million opioid users. In the same year, around 39.5 million individuals were living with drug use disorders. While most individuals with opioid dependence use illicitly produced heroin, the proportion of those using prescription opioids is increasing [7].

Studies conducted in Brazil indicate a marked increase in opioid use in recent years. Krawczyk et al. [8] reported a 465% rise in opioid sales between 2009 and 2015, while de Castro et al. [9] identified over 60 million opioid units dispensed between 2014 and 2018, predominantly codeine and tramadol, which accounted for about 95% of prescriptions. More recently, Correia et al. [10] observed a 34.8% increase in prescriptions between 2015 and 2020, along with a rise in units dispensed per capita. Across all studies, São Paulo consistently recorded the highest dispensing rates.

As opioid sales, prescribing, and hospital use increase in Brazil, the number of individuals exposed to these medications also rises, potentially expanding opioid-related harms. In this context, suspected or confirmed cases of exogenous intoxication must be reported to the Sistema de Informações sobre Agravos de Notificação (SINAN—Notifiable Diseases Information System), a national surveillance system that captures cases attended in health services, including data on the toxic agent, exposure circumstances, clinical management, and outcomes [11,12]. Data from SINAN are publicly available through the Departamento de Informática do Sistema Único de Saúde (DATASUS—Department of Informatics of the Unified Health System) and represent an important source of epidemiological information, supporting risk assessment and the characterization of health profiles across regions in Brazil [13]. Thus, increasing opioid availability is epidemiologically relevant not only as an indicator of greater access and use but also because it may lead to a higher number of intoxication events recorded in the surveillance system.

Despite the documented increase in opioid sales and prescribing in Brazil, the epidemiological implications of these trends remain incompletely characterized. Unpublished data from our research group indicate the clinical relevance of opioid-related harms, with opioid-related adverse events in a pediatric hospital involving mainly fentanyl, predominantly affecting children under 2 years of age, and presenting primarily with respiratory and cardiorespiratory complications, often in intensive care settings. However, these findings are limited to a single-center experience and do not reflect the broader epidemiological landscape of opioid-related intoxications in Brazil. In this context, national surveillance data are essential to better characterize the magnitude, distribution, and profile of opioid poisonings in the country.

We hypothesized that opioid-related poisoning notifications in Brazil increased between 2019 and 2025 and that these cases would present heterogeneous epidemiological, clinical, spatial, and temporal patterns, reflecting differences in opioid availability, exposure circumstances, co-exposures, healthcare access, and reporting practices across the country. Based on this hypothesis, this study aimed to compile and characterize opioid-related intoxication notifications recorded in SINAN, accessed through DATASUS, addressing the absence of consolidated national data and the limited number of previous studies using this source to understand the epidemiological scenario of opioid-notified poisonings in Brazil.

## 2. Results

The total number of notified poisonings due to medications and drugs of abuse between 2019 and 2025 was 1,127,265. Intoxication related to opioids occurred in 12,645 cases of notified poisoning. Table 1 shows the description of the number of notified poisonings of medications and drugs of abuse, and opioid-related poisonings per year. The annual distribution of notified poisonings showed an initial decrease in 2020, followed by a progressive increase in subsequent years. Notifications involving medications and drugs of abuse decreased from 126,958 in 2019 to 97,478 in 2020, then increased steadily from 2021 onward, reaching 276,290 in 2025. Opioid-notified poisonings followed a similar pattern, decreasing from 2063 cases in 2019 to 977 in 2020, followed by a year-by-year increase to 2875 cases in 2025. Overall, opioid-related notifications increased nearly threefold between 2020 and 2025.

Table 2 presents the main characteristics of intoxication cases and individuals involved in opioid-related poisonings. The opioids most frequently implicated in notifications were Codeine (4887; 38.7%), Tramadol (4297; 343.0%), Morphine (2259; 17.9%), Methadone (672; 5.3%), and Naltrexone (437; 3.5%). The majority of cases occurred in individuals aged 26–50 years (47.8%) and 0–25 years (39.3%). The median age was 32 years, and the mean ± SD age was 34.81 ± 15.91 years.

Most cases occurred in women (70.3%), and individuals recorded as white (56.0%), following the official categories used in Brazilian demographic records. Educational level was most frequently recorded as missing or not applicable (39.4%); the most common category was completed high school (22.4%). A large proportion of individuals were unemployed (12.9%), while a substantial proportion of the data was missing or ignored (44.6%). Most intoxications occurred in residences (85.6%). The primary route of exposure was digestive (92.3%), and the main circumstance was a suicide attempt (73.5%). Exposure types were heterogeneous, with acute-single (63.6%), acute-repeated (18.5%), chronic (2.0%), and acute-on-chronic (1.5%) patterns observed. Most patients were managed in hospital settings (78.4%), and nearly half were hospitalized (49.0%). Most patients recovered without sequelae (75.1%), while 1.6% died due to intoxication. The largest proportion of cases was reported in the Southeast region (47.0%), with the majority in the state of São Paulo (30.8%). Heroin-related notifications represented a small proportion of all opioid-related poisoning records and are described in the Supplementary Material, Section S1. Heroin, and Supplementary Table S1.

### 2.1. Co-Exposure Profile

Among the records with reported co-exposure information, patterns were analyzed at the substance level (Supplementary Table S2). Of the 9573 (75.7%) records with sufficient information for classification, co-exposure most frequently involved opioids and medications (8311; 86.8%), followed by multiple opioids plus medications (459; 4.8%), opioids with alcohol and medications (190; 2.0%), multiple opioids (160; 1.7%), and opioids and other illicit drugs (100; 1.0%), among others co-exposure substances identified with less than 1%.

For the co-exposure group, a separate descriptive analysis \was performed for the 482 cases classified as abuse. Substances reported across these fields were harmonized into categories and counted once per case, regardless of repeated mention in more than one field. Using this combined field approach, all abuse cases had at least one identifiable reported substance. One hundred and forty (29.0%) involved a single reported substance, whereas 342 (71.0%) involved two or more reported substances. The most frequently reported substances were codeine (121; 25.1%), morphine (109; 22.6%), tramadol (85; 17.6%), cocaine (77; 16.0%), alcohol (66; 13.7%), heroin (66; 13.7%), and fentanyl (42; 8.7%).

### 2.2. Comparative and Multivariable Analyses

Comparative and multivariable analyses were performed among eligible opioid-notified poisoning cases involving habitual use, abuse, or suicide attempts, with final classification as confirmed intoxication, exposure, or adverse reaction. Regarding the models, which have been restricted to records with complete information for the variables included and to the specific outcome definition under evaluation, denominators varied across analyses.

### 2.3. Suicide Attempt Versus Other Eligible Circumstances

A bivariate analysis was performed with eligible cases with suicide attempt involving habitual use, or abuse, with final classification as confirmed intoxication, exposure, or adverse reaction (Supplementary Table S3). In total, 9426 records were analyzed, of which 8573 (90.9%) were categorized as suicide attempts and 853 (9.1%) as other eligible circumstances (confirmed intoxication, exposure, or adverse reaction). Sex (*p* < 0.001), place of exposure (*p* < 0.001), co-exposure status (*p* < 0.001), and clinical outcome (*p* < 0.001) were significantly associated with the type of circumstance. Suicide attempts were more frequent among females than males (73.1% vs. 54.5%), and cases occurring in the residence were more common among suicide attempts than among other eligible circumstances (90.0% vs. 74.3%). Co-exposure was also more frequent among suicide attempts (82.3% vs. 70.6%), and cure without sequelae accounted for a higher proportion of cases among suicide attempts than among other eligible circumstances (83.5% vs. 74.9%). No significant differences were identified in employment status (*p* = 0.342) or hospitalization (*p* = 0.432).

In a contingency table analysis with Pearson’s chi-square test of the official Brazilian self-classified race/skin color categories, no statistically significant difference was observed in the distribution of suicide attempt versus other eligible circumstances (χ^2^ = 3.21; df = 5; *p* = 0.667; Fisher’s exact test *p* = 0.575). Suicide attempts represented the majority of cases across all race/skin color categories, ranging from 84.6% among Indigenous individuals to 92.1% among Black individuals (Supplementary Table S4).

In the multivariable logistic regression model evaluating factors associated with suicide attempt among eligible opioid-notified poisoning cases, female sex, residence-based exposure, co-exposure, younger age, and earlier notification year remained associated with suicide attempt after adjustment (Table 3). Females had higher odds of suicide attempt than males (aOR = 1.98; 95% CI: 1.68–2.34; *p* < 0.001). Exposures occurring in residences were strongly associated with higher odds of suicide attempt compared with exposures occurring in external environments (aOR = 8.95; 95% CI: 6.29–12.72; *p* < 0.001). Cases with co-exposure had higher odds of being classified as suicide attempts compared with cases without co-exposure (aOR = 2.17; 95% CI: 1.81–2.60; *p* < 0.001). In contrast, increasing age was associated with lower odds of suicide attempt, with a 3.9% decrease in odds for each additional year of age (aOR = 0.961; 95% CI: 0.955–0.966; *p* < 0.001). Year of notification was also inversely associated with suicide attempt (aOR = 0.919; 95% CI: 0.884–0.956; *p* < 0.001).

### 2.4. Hospitalization Outcome

The hospitalization analysis was restricted to eligible cases included in the comparative analysis, after excluding records that did not meet the predefined eligibility criteria (habitual use, abuse, or suicide attempts, with final classification as confirmed intoxication, exposure, or adverse reaction) or had missing hospitalization status. Therefore, the denominator differs from that used in the overall descriptive analysis presented in Table 2, which included all 12,645 opioid-notified poisoning notifications.

A bivariate analysis was performed among 9160 eligible cases with non-missing hospitalization status (Supplementary Table S5), 4853 (53.0%) were hospitalized, and 4307 (47.0%) were not hospitalized. Sex (*p* = 0.002), co-exposure status (*p* = 0.033), and clinical outcome (*p* < 0.001) were significantly associated with hospitalization status. Hospitalized cases showed a different sex distribution compared with non-hospitalized cases, with males representing a slightly higher proportion among hospitalized patients (30.0% vs. 27.1%). Co-exposure was also more frequent among hospitalized than non-hospitalized cases (82.0% vs. 80.2%). In addition, other outcomes were proportionally more frequent among hospitalized than among non-hospitalized cases (22.3% vs. 10.7%). No significant differences were identified in the employment status (*p* = 0.225), place of exposure (*p* = 0.851), or circumstance of exposure (*p* = 0.432). Considering only residence and external environment, residence accounted for the vast majority of cases in both groups (98.2% among hospitalized and 98.1% among non-hospitalized cases). In contrast, exposures in external environments were uncommon (1.8% and 1.9%, respectively).

In a contingency table analysis with Pearson’s chi-square test of official Brazilian self-classified race/skin color categories, hospitalization status differed across categories (χ^2^ = 17.3; df = 5; *p* = 0.004). Hospitalization was most frequent among records classified as ignored/missing for race/skin color (57.9%), followed by White individuals (53.4%) and Brown/Parda individuals (52.4%). Lower proportions were observed among Black individuals (45.3%) and Indigenous individuals (38.5%), although the Indigenous category included very small numbers and should be interpreted cautiously (Supplementary Table S6).

In the multivariable logistic regression model evaluating hospitalization among eligible opioid-notified poisoning cases, sex and co-exposure status remained associated with hospitalization after adjustment (Table 3). Females had lower odds of hospitalization than males (aOR = 0.83; 95% CI: 0.75–0.91; *p* < 0.001). Cases with co-exposure also had higher odds of hospitalization compared with cases without co-exposure (aOR = 1.14; 95% CI: 1.02–1.27; *p* = 0.025). Age, year of notification, and place of exposure were not associated with hospitalization after adjustment.

### 2.5. Factors Associated with Outcomes

Comparative eligible cases included those involving habitual use, abuse, or suicide attempts, with final classification as confirmed intoxication, exposure, or adverse reaction. In total, 9426 records were analyzed; 91.0% of cases were categorized as suicide attempts, 5.1% as abuse, and 3.9% as habitual use, with 7533 cases classified as cured without sequelae and 1568 as other outcomes (cure with sequelae, death due to exogenous intoxication, death due to another cause, loss to follow-up, unknown).

A bivariate analysis was performed with the eligible cases of the type of outcome (Supplementary Table S7). Exposures occurring in residences were more frequent among cases with a cure without sequelae than among those with other outcomes (98.6% vs. 96.3%; *p* < 0.001). A significant difference was also identified in the female sex among cases with a cure without sequelae than among those with other outcomes (72.1% vs. 69.2%; *p* = 0.024). No significant differences were identified for employment status (*p* = 0.673) or co-exposure status (*p* = 0.638).

In a contingency table analysis with Pearson’s chi-square test of official Brazilian self-classified race/skin color categories, the distribution of cure without sequelae versus other outcomes differed across categories (χ^2^ = 79.4; df = 5; *p* < 0.001). Cure without sequelae was the most frequent outcome across all self-classified race/skin color categories, with the highest proportions observed among White individuals (84.3%), followed by Brown (82.9%), Black (81.6%), Yellow/Asian (80.3%), and Indigenous individuals (75.0%). Records classified as ignored/missing for race/skin color showed the lowest proportion of cure without sequelae (69.5%). The proportion of other outcomes was highest among records classified as ignored/missing for race/skin color (30.5%). Among valid self-classified race/skin color categories, the proportion of other outcomes ranged from 15.7% among White individuals to 25.0% among Indigenous individuals, although the Indigenous category included very small numbers and should be interpreted cautiously (Supplementary Table S8).

In the multivariable logistic regression model evaluating other outcomes (cure with sequelae, death due to exogenous intoxication, death due to another cause, loss to follow-up, unknown) versus cure without sequelae, age, year of notification, place of exposure, and circumstance of exposure remained associated with other outcomes after adjustment (Table 3). Increasing age was associated with slightly higher odds of other outcomes (aOR = 1.006; 95% CI: 1.001–1.010; *p* = 0.009), and year of notification was also positively associated with other outcomes (aOR = 1.053; 95% CI: 1.024–1.084; *p* < 0.001). Exposures occurring in residences were associated with lower odds of other outcomes compared with exposures occurring in external environments (aOR = 0.397; 95% CI: 0.277–0.569; *p* < 0.001). Suicide attempts were also associated with lower odds of other outcomes compared with other eligible circumstances (aOR = 0.749; 95% CI: 0.610–0.921; *p* = 0.006). Sex and co-exposure status were not associated with other outcomes after adjustment.

### 2.6. Serious Outcomes Versus Cure Without Sequelae

Serious outcomes were defined as recovery with sequelae, death due to exogenous intoxication, or death due to another cause (Supplementary Table S9). A bivariate analysis was performed among 7876 eligible cases with classifiable outcomes for this comparison; 343 (4.4%) had serious outcomes, and 7533 (95.6%) recovered without sequelae. Serious outcomes were significantly associated with sex (*p* = 0.009), place of exposure (*p* ≤ 0.001), co-exposure status (*p* = 0.001), hospitalization (*p* < 0.001), and circumstance (*p* < 0.001), but not with employment status (*p* = 0.310). Considering only residence and external environment, the place of exposure remained significantly associated with outcome (*p* < 0.001). Residence accounted for most cases in both groups, although it was proportionally less frequent among serious outcomes than among cases with a cure without sequelae (94.4% vs. 98.6%). Conversely, exposures occurring in external environments were proportionally more frequent among serious outcomes (5.6%) than among cases with a cure without sequelae (1.4%).

In a contingency table analysis with Pearson’s chi-square test of official Brazilian self-classified race/skin color categories, no statistically significant difference was observed in the distribution of serious outcomes versus cure without sequelae (χ^2^ = 1.91; df = 5; *p* = 0.861; Fisher’s exact test *p* = 0.777). Serious outcomes were infrequent across all categories, ranging from 1.6% among Yellow/Asian individuals to 10.0% among Indigenous individuals (Supplementary Table S10).

In the multivariable logistic regression model evaluating serious outcomes versus cure without sequelae, age, place of exposure, and circumstance of exposure remained associated with serious outcomes after adjustment (Table 3). Exposures occurring in residences were associated with lower odds of serious outcomes compared with exposures occurring in external environments (aOR = 0.301; 95% CI: 0.167–0.543; *p* < 0.001). Suicide attempts were also associated with lower odds of serious outcomes compared with other eligible circumstances (aOR = 0.441; 95% CI: 0.314–0.620; *p* < 0.001). Age was associated with serious outcomes, with a 1.7% increase in the odds of serious outcomes for each additional year of age (aOR = 1.017; 95% CI: 1.009–1.026; *p* < 0.001). Sex, co-exposure status, and year of notification were not associated with serious outcomes after adjustment.

### 2.7. Spatial and Temporal Trends

**Figure 1:**
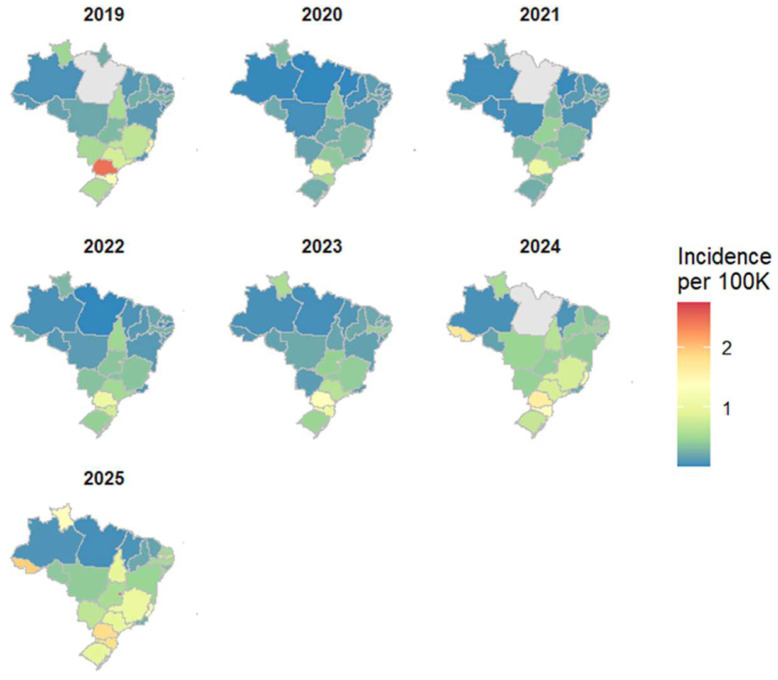
Spatial distribution of opioid poisoning incidence by federative unit in Brazil, 2019–2025. Choropleth maps representing the annual incidence of opioid poisonings (cases per 100,000 inhabitants) in Brazilian federative units from 2019 to 2025. Colors vary along a continuous scale from blue (lowest incidence) to red (highest incidence), allowing visualization of spatial gradients and temporal variations. Grey areas indicate missing data or unavailable records for the respective year. Source: DATASUS/SINAN and Instituto Brasileiro de Geografia e Estatística—IBGE (Brazilian Institute of Geography and Statistics).

The spatial analysis of opioid poisoning incidence in Brazil from 2019 to 2025 (Figure 1) shows a heterogeneous pattern across federative units, with variations over time. In general, the North and part of the Central-West regions showed low incidence levels throughout the entire period, predominantly represented by blue shades. This pattern suggests relative stability and a lower magnitude of the condition in these regions. In contrast, the South region and part of the Southeast had the highest incidence values. In 2019, one state in the South region stood out with a high incidence (red shades), indicating a possible initial peak. In subsequent years (2020–2023), these more extreme values decreased, with a predominance of intermediate incidence levels (green and yellow shades). From 2024 onward, and especially in 2025, an increasing trend in incidence was observed in several federative units, particularly in the South, Southeast, and part of the Northeast, as shown by the expansion of yellow and orange areas. This pattern suggests a possible spread or intensification of the problem across the national territory. Additionally, some states showed greater temporal variability, indicating fluctuations in incidence over the years, while others maintained more stable patterns. Finally, the presence of gray areas in certain years suggests missing data or inconsistencies in reporting, which should be taken into account when interpreting the results.

**Figure 2:**
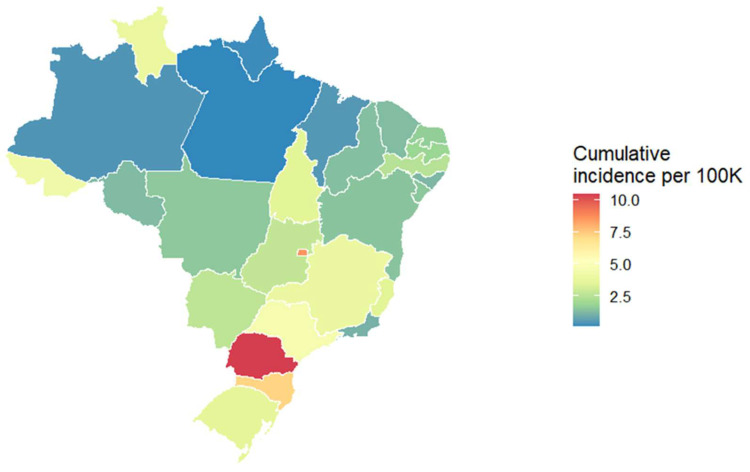
Cumulative incidence of opioid poisonings in Brazil, by federative unit, from 2019 to 2025. The map presents cumulative incidence rates per 100,000 inhabitants, calculated from the sum of reported cases over the period and the average state population. Data were obtained from DATASUS/SINAN and IBGE.

The analysis of cumulative opioid poisoning incidence in Brazil from 2019 to 2025 also reveals a heterogeneous spatial pattern across federative units (Figure 2). The highest cumulative incidence values were concentrated in the South region, with one state showing the highest value in the country (red shades), followed by adjacent states with intermediate to high incidence (orange and yellow shades). This pattern indicates a regional concentration of the condition over the analyzed period. In the Southeast region, intermediate values predominated, with variation among states, suggesting a moderate burden of the event. The Central-West region generally showed low to moderate incidences, with relative spatial homogeneity. The North and part of the Northeast regions showed the lowest cumulative incidence values, represented by blue shades, indicating a lower magnitude of the condition over the period. However, some states in the Northeast exhibited intermediate values, suggesting intraregional heterogeneity.

### 2.8. Predictive Models

**Figure 3:**
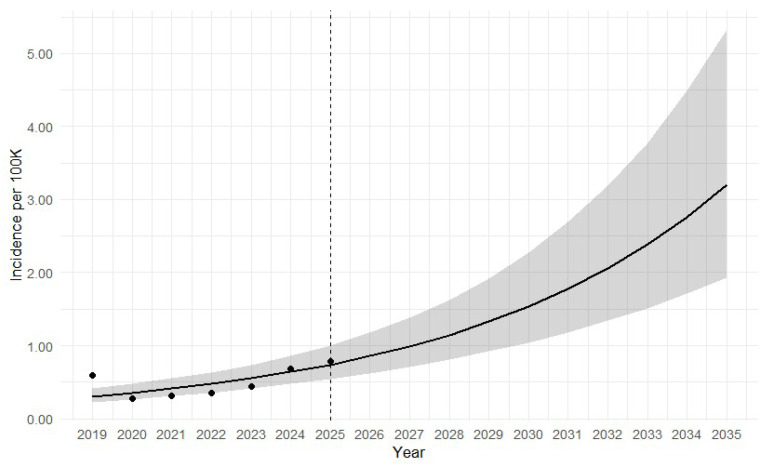
Observed and predicted incidence of opioid poisoning in Brazil. Note: Negative binomial mixed model with 95% CI. An increasing trend in opioid poisoning incidence in Brazil is observed over the analyzed period. The points represent the observed values between 2019 and 2025, while the solid line indicates the incidence estimated by the negative binomial mixed model. The shaded area corresponds to the 95% confidence interval of the estimates. The vertical dashed line marks the beginning of the projection period (after 2025), during which a progressive increase in incidence is observed, accompanied by widening confidence intervals, reflecting greater uncertainty in future estimates.

A Poisson mixed model and a negative binomial mixed model were fitted to assess the temporal trend in opioid poisoning cases in Brazil, considering the logarithm of the population as an offset and a random intercept for each federative unit. A significant effect of year was observed in both models (*p* < 0.001), indicating a temporal increase in cases. In the Poisson model, the coefficient associated with year was 0.110 (SE = 0.006), whereas in the negative binomial model it was 0.145 (SE = 0.016). Comparison between models showed better fit for the negative binomial model (AIC = 1265.8) compared with the Poisson model (AIC = 1802.5), which also showed overdispersion, with phi = 8.04. Variability between states was consistent in both models, with the standard deviation of the random intercept close to 0.88 (Supplementary Table S11). The negative binomial model suggests that the rate of opioid poisonings increased, on average, by approximately 15.6% per year during the analyzed period, after adjustment for population size and variability between states (Supplementary Table S12). The evolution of opioid poisoning incidence can be better visualized in Figure 3 and Figure S1.

## 3. Discussion

The present findings indicate that opioid-notified poisonings in Brazil already constitute a relevant and nationally distributed epidemiological burden rather than isolated events. Across 12,645 reported cases identified between 2019 and 2025, notifications increased in the most recent years of the series, with 2024 and 2025 accounting for the largest annual numbers. The profile observed was marked by a predominance of females, adults aged 26–50 years, and residents of the Southeast and South regions, especially São Paulo and Paraná, suggesting that reported opioid-notified poisonings were more frequently captured in more populous and highly connected settings, where opioid circulation, healthcare access, and notification capacity may be greater. Spatial differences should also be interpreted with caution, as lower rates in the North, Northeast, or parts of the Central-West may reflect underdetection or underreporting rather than a genuinely lower burden. Thus, the spatial findings are best interpreted as the geographic distribution of reported opioid-notified poisonings within SINAN/DATASUS, not as a direct measure of the underlying true incidence across Brazilian regions.

The predominance of suicide attempts and digestive exposure indicates that opioid-notified poisonings captured in SINAN/DATASUS are strongly linked to intentional ingestion and household medication availability, rather than to therapeutic use or recreational use alone. Co-exposure was frequent, particularly with other medications, reinforcing the need to interpret these events within broader medication-use and self-poisoning contexts.

The most frequent poisoning profile was not necessarily the profile associated with the worst outcomes. Overall, opioid-notified poisonings were more commonly reported among females and were predominantly linked to residence-based exposures and suicide attempts. However, the profile associated with severity differed from the most frequent notification profile. In the bivariate analysis, serious outcomes were proportionally more frequent among males, hospitalized cases, cases with co-exposure, and exposures occurring outside the residence. After adjustment, however, serious outcomes remained independently associated with older age, while residence-based exposures and suicide attempts were associated with lower odds of serious outcomes compared with external environments and other eligible circumstances, respectively. This suggests that the epidemiological profile of occurrence should not be interpreted in the same way as the profile of severity.

Rather, the findings point to partially overlapping patterns: a more common profile characterized by intentional ingestion in domestic settings, and a less frequent but potentially more severe profile associated with different exposure contexts and clinical trajectories. This interpretation is consistent with recent Brazilian studies on poisoning and suicide attempts, which also reported a predominance of female cases, household exposure, and generally favorable outcomes among the most frequent notifications [14,15].

The increase in reported cases after 2023 should be interpreted as a multifactorial and exploratory finding rather than as evidence of a single causal mechanism. Several non-mutually exclusive explanations may have contributed to this pattern, including changes in surveillance sensitivity and reporting practices, particularly after updated national regulations, standardization of weekly reporting, and the issuance of technical notes and local investigation protocols, especially in São Paulo [16,17]. Post-pandemic reorganization of healthcare access and notification flows may also have influenced case detection, as previous Brazilian evidence showed a reduction in reported medication-related exogenous intoxications during the first and second years of the COVID-19 pandemic, possibly reflecting surveillance disruption and underreporting [18]. In addition, real changes in exposure or toxicological risk cannot be excluded, particularly in the context of drugs of abuse and the circulation of synthetic substances, including synthetic cannabinoids and emerging synthetic opioids [19,20,21]. However, the present ecological and surveillance-based design does not allow the relative contribution of these factors to be quantified. Therefore, the observed increase should not be interpreted as reflecting a true rise in opioid poisoning incidence alone, but rather as a signal that may combine epidemiological changes with improvements or fluctuations in case detection and reporting.

Although the temporal model projected a continued increase in opioid poisoning incidence up to 2035, these estimates should be interpreted with caution. Rather than definitive forecasts, the projections represent exploratory and hypothetical scenarios based on the continuation of the temporal pattern observed in the available data. This distinction is particularly important because the fitted trend is strongly influenced by the marked increase in notifications observed in 2024 and 2025. Moreover, future opioid poisoning incidence may follow non-linear dynamics that cannot be fully captured from a relatively short observational period. Regulatory changes, modifications in prescribing and dispensing practices, shifts in illicit drug markets, and the emergence of highly potent synthetic opioids may either intensify or attenuate future trends. Therefore, the projections should be interpreted as scenario-based estimates that highlight a potential public health concern, rather than as precise predictions of the future burden of opioid-notified poisonings in Brazil.

The predominance of suicide attempts deserves particular emphasis, as it indicates that the Brazilian notification profile captured in SINAN is strongly shaped by intentional self-poisoning rather than by recreational opioid use alone or by therapeutic adverse events in isolation. In this sense, the current findings suggest that the national burden captured by the notification system may be more closely intertwined with self-harm and psychiatric vulnerability than with the classic North American pattern of community overdose driven primarily by illicit opioid use [22,23,24].

The multivariable model further supported this interpretation, showing that suicide attempts were independently associated with female sex, younger age, residence-based exposure, and co-exposure. These findings reinforce the interpretation that many opioid-notified poisonings captured by SINAN/DATASUS occur in a context of intentional self-poisoning, often involving medications available in the household and multiple substances. The inverse association with notification year suggests that, although suicide attempts remained predominant, other exposure circumstances may have become relatively more visible in more recent notifications.

The inclusion of self-classified race/skin color in this analysis is also relevant from a public health surveillance perspective. In Brazil, this variable should not be interpreted as a biological determinant, but as a social and administrative marker that may reflect structural differences in access to healthcare, continuity of care, medication availability, prescription pathways, and reporting practices. This interpretation is supported by national evidence showing that White Brazilians reported better access to healthcare services and medications than Black and Brown Brazilians, with the largest inequalities observed for unmet healthcare needs and access to medication; these differences were largely explained by socioeconomic factors, particularly income and access to private health insurance [25]. In addition, Instituto Brasileiro de Geografia e Estatística—IBGE (Brazilian Institute of Geography and Statistics) reports on social inequalities by color or race, reinforcing the use of this category as an important marker for monitoring structural inequalities in Brazil [26]. This is particularly important in the context of intentional self-poisoning, since the ingestion of opioids and multiple medications implies previous access to these substances through some healthcare, household, or informal source. Therefore, examining self-classified race/skin color may help identify potential social differences in exposure contexts, access to medicines, and interaction with healthcare and surveillance systems, while avoiding any interpretation of race/skin color as a causal or biological explanation for poisoning risk.

Another aspect that can influence both the management and quality of reporting is the limitation of training in clinical toxicology and applied pharmacology [27]. Gaps in the recognition of active principles, pharmacological classes, potential interactions, signs of toxicity, and reporting criteria can delay diagnosis, hinder the correct classification of the agent involved, and compromise surveillance [28]. This point is particularly relevant in the context of opioids and other psychoactive substances, where differences in formulations, potency, toxicological profile, and regulatory requirements have important clinical and epidemiological implications [29,30].

Most classifiable records involved opioids in combination with other medications, and co-exposure remained independently associated with both suicide attempts and hospitalization. However, co-exposure did not remain independently associated with other outcomes versus cure without sequelae or with serious outcomes after adjustment. Taken together, these findings suggest that co-exposure should not be interpreted as a uniformly aggravating factor. Its clinical significance likely depends on the specific substances involved, the amount ingested, the route of exposure, and the behavioral context in which poisoning occurred. This interpretation is compatible with recent literature indicating that the risks associated with opioid-notified polysubstance use depend strongly on the specific combinations involved, particularly when opioids are combined with stimulants such as cocaine [31].

In Brazil, opioid prescribing and dispensing requirements vary by substance and formulation, which may influence access, circulation, and poisoning patterns. Although opioid access remains relatively restrictive compared with high-income settings, codeine and tramadol are more familiar in outpatient practice and may circulate more broadly than stronger opioids (morphine, metadone and fentanyl) used mainly in hospital, severe pain, or palliative care contexts [4,32,33]. This may help explain why codeine, tramadol, and morphine were the most frequently reported opioids in the present study, while recent alerts involving fentanyl and nitazenes reinforce the importance of surveillance for emerging synthetic opioids [21,34].

These findings should be interpreted alongside, but not directly compared with, opioid dispensing studies. Américo et al. [35], using outpatient SUS data from 2018 to 2023, reported codeine as the most dispensed opioid, followed by morphine and methadone, and observed a moderate annual increase in opioid dispensing. In the present study, the estimated annual increase in opioid poisoning notifications reflects a different outcome, denominator, and surveillance process. Dispensing indicators measure population-level access or consumption within a specific healthcare system, whereas poisoning notifications capture reported adverse, intentional, or toxic exposure events among individuals who reached health services and were notified in SINAN/DATASUS. Therefore, the higher annual increase estimated for poisoning notifications should not be interpreted as a direct consequence of, or as proportional to, opioid consumption trends. Rather, these findings suggest that opioid-notified poisoning notifications may increase through pathways that include, but are not limited to, greater availability, intentional self-poisoning, co-exposures, other recorded exposure circumstances, and changes in reporting sensitivity [36].

Although heroin represented only a small proportion of all opioid-notified notifications, its descriptive profile is still relevant as an exploratory finding. The small number of heroin-related records (105) limits the strength of inference and precludes definitive conclusions about changes in illicit drug market dynamics. Nevertheless, the predominance of male cases, abuse-related circumstances, and frequent polysubstance co-reporting may represent a potential epidemiological signal that warrants closer monitoring in future studies. Therefore, heroin-related notifications should be interpreted not as evidence of a quantitatively dominant burden, but as a small and exploratory subgroup that may help identify emerging patterns of mixed psychoactive substance exposure within the Brazilian notification system. This low number should also be interpreted in light of the scope of SINAN/DATASUS, which captures medically attended and notified poisoning cases rather than forensic deaths, deaths occurring before healthcare access, untreated intoxications, or cases not reported to the health surveillance system. Therefore, heroin-related notifications in this dataset may underestimate the broader burden of heroin-related harm in Brazil [1,21,37].

Taken together, these findings suggest that opioid-notified poisonings in Brazil should be interpreted as a heterogeneous phenomenon involving at least three partially overlapping scenarios: first, a dominant pattern of intentional domestic ingestion, strongly linked to suicide attempts; second, a clinically relevant group of cases associated with hospitalization and more severe outcomes; and third, a smaller but strategically important subset of notifications reflecting abuse, heroin involvement, and polysubstance exposure. Importantly, although suicide attempts constituted the predominant exposure circumstance, the notification profile captured by SINAN/DATASUS should not be interpreted exclusively as a self-poisoning dataset. Rather, it reflects a broader toxicovigilance scenario encompassing multiple exposure contexts, including abuse, habitual use, adverse reactions, unintentional exposures, and polysubstance poisonings.

From a public health and policy perspective, these findings support the need to strengthen opioid-notified toxicovigilance in Brazil. In addition, small exploratory signals involving heroin and synthetic opioids reinforce the importance of early warning systems and intersectoral monitoring involving health surveillance, toxicology services, forensic laboratories, and drug policy authorities.

## 4. Materials and Methods

This study is a retrospective, descriptive investigation based on active retrieval of relevant information from publicly available, open-access databases. Because the analyses were conducted exclusively using secondary, aggregated, and/or de-identified data in the public domain, with no direct contact with participants, approval from a Research Ethics Committee was not required, in accordance with applicable regulations.

### 4.1. Data Access

The data were accessed through the DATASUS platform, which provides freely accessible data from official Sistema Único de Saúde (SUS—Unified Health System) system databases, including SINAN data reporting exogenous poisonings between 2019 and 2025 across the entire Brazilian territory. This system includes different categories of compulsory notification; only records classified as exogenous intoxication related to medications and drugs of abuse were included in this study. Data for 2019–2023 were accessed in March 2024, whereas data for 2024–2025 were accessed in February 2026. Therefore, the most recent years may remain subject to revision and database updating.

The data were collected using the SINAN Net data dictionary, version 5.0, which provides detailed guidelines for completing all fields, including the use of numerical codes or abbreviations and their corresponding meanings. Data were qualified and standardized before analysis. Records referring to the same substance or variable but reported with different spellings, abbreviations, or nomenclature were harmonized into standardized categories to ensure internal consistency and enable reliable descriptive analyses. The information contained in this dictionary was also used to translate and standardize variables using R software, version 4.3.3 (R Core Team, 2024) [38], a free and open-source tool for statistical computing.

Sex was analyzed as recorded in the SINAN notification form and corresponds to the biological sex documented at the time of notification. Race/skin color was analyzed as recorded in the SINAN database and corresponds to the official Brazilian self-classified “color or race” categories used in national health and demographic information systems. These categories were translated as White, Black, Brown, Yellow/Asian, and Indigenous. Race/skin color categories were interpreted as social and administrative self-classification categories, not as biological or genetic ancestry categories.

### 4.2. Inclusion Criteria and Case Identification

From the general SINAN/DATASUS dataset, records classified as exogenous poisonings involving medications or drugs of abuse were first selected. Opioid-notified poisoning notifications were then identified by screening substance fields for opioid analgesic active ingredients, reference names, commercial names, and opioid-notified terms listed in the Brazilian Thesaurus of Medicines. The screening list containing their reference name, commercial name, and active ingredient, screened according to the following groups: buprenorphine (restiva, transtec), codeine (paco, codein, tylex, codex), fentanyl, heroin, hydrocodone, methadone (mytedom), morphine (dimorf), nalbuphine (nubain), naloxone, naltrexone (revia, uninaltrex), opioid (opiaceo), opium, oxycodone (oxycontin), tapentadol (palexis), tramadol (tramal, revange, paratram, ultracet).

### 4.3. Co-Exposure Classification

When multiple substances were reported in a single notification, all exposures were recorded. Co-exposure information was subsequently extracted through manual, line-by-line review of each record to capture all substances reported in free-text or non-standardized fields. Each co-exposure entry was then classified according to the following predefined categories: (1) opioids and medications, (2) multiple opioids plus medications, (3) opioids with alcohol and medications, (4) multiple opioids, and (5) opioids with drugs. Another classification used is presented in Supplementary Table S2. The formulations that combine some opioids with acetaminophen were also considered a poisoning associated with another medication.

### 4.4. Statistical Analysis

Statistical analyses were performed using Jamovi (version 2.6.44) and R (version 4.3.3; R Core Team, 2024) [38]. Continuous variables were summarized as mean and standard deviation. Categorical variables were presented as absolute and relative frequencies (N, %). Statistical significance was set at *p* < 0.05.

### 4.5. Bivariate Analysis

Comparative bivariate analyses were conducted to explore crude associations between selected demographic, exposure-related, and clinical variables and predefined clinically or epidemiologically relevant outcomes among eligible opioid-notified poisoning cases. Eligible cases included notifications involving habitual use, abuse, or suicide attempts, with final classification as confirmed intoxication, exposure, or adverse reaction. Four main bivariate comparisons were performed: cure without sequelae versus other outcomes (cure with sequelae, death due to exogenous intoxication, death due to another cause, loss to follow-up, unknown); suicide attempt versus other eligible circumstances (habitual use, or abuse, with final classification as confirmed intoxication, exposure, or adverse reaction); hospitalization versus no hospitalization; and serious outcomes (recovery with sequelae, death due to exogenous intoxication, or death due to another cause) versus cure without sequelae.

Clinical outcomes were defined according to the outcome categories available in SINAN/DATASUS, including hospitalization status, recovery without sequelae, recovery with sequelae, death due to exogenous intoxication, and death due to other causes.

For each comparison, categorical variables such as sex, employment status, place of exposure, co-exposure status, circumstance of exposure, hospitalization status, clinical outcome, and self-classified race/skin color category were evaluated when applicable and available. Results were presented as absolute and relative frequencies within each comparison group, and differences between groups were assessed using Pearson’s chi-square test or Fisher’s exact test when expected cell counts were low (less than 5).

These bivariate analyses were exploratory and descriptive, intended to identify unadjusted differences between groups rather than independent associations. Records with missing or non-classifiable information for the variables required in each specific comparison were excluded from that analysis, resulting in different denominators across comparative tables.

### 4.6. Multivariable Binary Logistic Regression

Multivariable binary logistic regression models were fitted to assess factors associated with selected outcomes among eligible opioid-notified poisoning cases. Eligible cases followed the same criteria used in the bivariate analyses, including notifications involving habitual use, abuse, or suicide attempts, with final classification as confirmed intoxication, exposure, or adverse reaction. Four separate logistic regression models were fitted: other outcomes (cure with sequelae, death due to exogenous intoxication, death due to another cause, loss to follow-up, unknown) versus cure without sequelae; suicide attempt versus other eligible circumstances (habitual use, or abuse, with final classification as confirmed intoxication, exposure, or adverse reaction); hospitalization versus no hospitalization; and serious outcomes (recovery with sequelae, death due to exogenous intoxication, or death due to another cause) versus cure without sequelae.

Age and year of notification were included as continuous variables. Sex, place of exposure, co-exposure status, and circumstance of exposure were included as categorical covariates according to the specific outcome evaluated in each model. Hospitalization and clinical outcome were not included as predictors in the suicide attempt model because they occur after the poisoning circumstance and may represent downstream consequences of the event. Similarly, hospitalization was not included as a predictor in the serious outcome model because it may reflect clinical severity or healthcare management after the poisoning event rather than an exposure preceding the outcome. All logistic regression analyses were conducted using complete-case analysis, and records with missing or non-classifiable values for any variable included in each model were excluded from the corresponding analysis. Results were reported as adjusted odds ratios (aORs), 95% confidence intervals, and *p*-values.

### 4.7. Incidence Analysis

The incidence of opioid poisoning was calculated as the number of cases divided by the corresponding annual population, multiplied by 100,000 inhabitants. For the period from 2019 to 2025, cumulative incidence by state was estimated. To do so, the total number of cases over the period was summed, and the average population was used as the denominator. Choropleth maps were created using geospatial data from the Brazilian Federative units, allowing visualization of the spatial distribution of incidence over time.

To assess the temporal trend in incidence and account for the hierarchical structure of the data (observations nested within states), generalized linear mixed models were fitted. Models with Poisson and negative binomial distributions were fitted, using the number of cases as the dependent variable. Year was included as a fixed effect, the state-level intercept as a random effect, and the logarithm of the population as an offset. This offset allowed the model to account for differences in population size across federative units and over time, including changes in the Brazilian population during the study period based on IBGE population estimates. Therefore, the temporal trend and projected incidence estimates were adjusted for population growth rather than being based only on absolute case counts.

$$\log\left(E\left[Y_{ij}\right]\right)=\beta_{0}+\beta_{1}\left({year}_{ij}\right)+\mu_{j}+log({population}_{ij})$$

The model specification was:

**where**

- **Y_ij_** represents the number of cases in state (*j*) in year (*i*), and
- **µ_i_** represents the random effect of the state.

Overdispersion was assessed using the ratio of Pearson residuals to degrees of freedom. Given the evidence of overdispersion (phi > 1), the alternative negative binomial model was used and selected based on the Akaike Information Criterion (AIC). In addition, a zero-inflated negative binomial model with population offset was also fitted to assess whether accounting for excess zeros would improve model performance. However, this alternative specification did not provide a statistically or substantively meaningful improvement in model fit and did not alter the interpretation of the temporal trend. Therefore, the negative binomial mixed model was retained as the most stable and parsimonious model for the main analysis. From the negative binomial model, predicted values for cases and incidence were obtained for the observed period.

For future projections up to 2035, the population of each state was estimated using simple linear regression over time based on IBGE population estimates. These projected populations were then incorporated into the model through the population offset, allowing future incidence estimates to account for expected population growth. Then, the predicted case values obtained from the negative binomial model were converted into incidence per 100,000 inhabitants. Ninety-five percent confidence intervals were calculated on the linear predictor scale (log link) and subsequently transformed back to the original scale. The results were aggregated at the national level by summing cases and populations by year, allowing estimation of the observed and predicted national incidence. Results were presented as time series with observed and predicted values, as well as 95% confidence intervals. Stratified time series by federative unit were also constructed.

### 4.8. Use of Artificial Intelligence-Assisted Tools

During the preparation of this manuscript, the authors used ChatGPT (OpenAI, GPT-5.5 Thinking) for English language editing and text refinement. The authors carefully reviewed and edited all AI-assisted content and take full responsibility for the final version of this publication.

## 5. Conclusions

This nationwide analysis of SINAN/DATASUS notifications indicates that opioid-notified poisonings in Brazil are not isolated events, but a heterogeneous and increasingly visible public health issue. The burden captured by the surveillance system was mainly characterized by intentional self-poisoning, household and digestive exposures, medication co-ingestion, and the frequent involvement of commonly reported opioids such as codeine, tramadol, and morphine. Importantly, the most frequent notification profile was not necessarily the profile associated with the greatest severity. Although serious outcomes were proportionally more frequent among men, hospitalized cases, and exposures occurring outside the residence in bivariate analyses, the multivariable model indicated that serious outcomes were independently associated with older age, whereas residence-based exposures and suicide attempts were associated with lower odds of serious outcomes compared with external environments and other eligible circumstances, respectively. The increase observed in recent years, as well as the exploratory projections, should be interpreted cautiously because they may reflect both changes in opioid-notified risk and variations in healthcare access, case detection, reporting sensitivity, and surveillance practices. Overall, these findings support the need to strengthen opioid-notified toxicovigilance in Brazil through improved notification quality, professional training in clinical toxicology, integration with suicide-prevention strategies, safer prescribing, dispensing, storage, and disposal practices, and early monitoring of polysubstance exposure and emerging synthetic opioids.

## 6. Limitations

As the dataset was retrieved from SINAN through DATASUS, underreporting, missing data, and regional differences in reporting capacity are possible. Recorded fields may also contain errors due to misclassification, transcription mistakes, or inconsistent documentation of medication names. In some records, the stated active principle was incongruent with the corresponding drug, and given the secondary nature of the data and the absence of validation mechanisms, it was not possible to determine whether such discrepancies reflected true involvement of an additional toxic agent or inaccuracies introduced during data entry or reporting.

Missing, ignored, and incomplete fields may have introduced selection bias, particularly in subgroup and bivariate analyses. No imputation was performed because the missingness mechanism could not be reliably determined from the surveillance database. Co-exposure classification also required manual harmonization of free-text and non-standardized fields, and formal inter-rater reliability measures were not available; therefore, co-exposure patterns should be interpreted cautiously. In addition, multiple bivariate comparisons were conducted without formal adjustment for multiple testing; therefore, statistically significant *p*-values from these exploratory analyses should be interpreted cautiously and considered hypothesis generating.

Although multivariable logistic regression models were fitted to adjust for selected covariates, these analyses remained exploratory and were limited by the observational design, complete-case approach, missing data, potential misclassification, and residual confounding. In addition, the temporal sequence between some clinical variables and outcomes cannot be fully established using SINAN/DATASUS records. This is particularly relevant for hospitalization, which may reflect clinical severity or healthcare management after poisoning rather than a causal determinant of serious outcomes. Therefore, the observed associations should not be interpreted as causal effects, but rather as hypothesis-generating evidence of potential relationships between demographic, exposure-related, and clinical variables and opioid poisoning outcomes.

The most recent SINAN/DATASUS records may still be subject to database consolidation, late entry, correction, and updating, and part of the observed increase may reflect changes in reporting completeness or data processing rather than a true epidemiological rise alone. This limitation is particularly relevant for the temporal models and projections, since the fitted trend is influenced by the most recent years of the series. Therefore, both the observed acceleration and the projected estimates should be interpreted as exploratory and provisional, and future analyses should reassess these patterns as updated datasets become available.

The SINAN/DATASUS database does not provide information regarding whether the opioid involved was prescribed to the exposed individual, obtained through informal sources, or accessed through other pathways. Therefore, the present study cannot determine the proportion of poisonings directly attributable to prescribed opioid use. Detailed clinical severity indicators (e.g., opioid dose, laboratory findings, respiratory support requirements, intensive care admission, or standardized poisoning severity scores) were not available in the surveillance database.

## Figures and Tables

**Table 1:** Annual distribution of reported poisoning cases involving medications, drugs of abuse, and opioids recorded in SINAN/DATASUS, Brazil, 2019–2025.

Year	Notified Poisonings of Medications and Drugs of Abuse n (%)	Opioid-Notified Poisonings n (%)
2019	126,958 (11.26)	2063 (16.31)
2020	97,478 (8.65)	977 (7.73)
2021	102,999 (9.14)	1118 (8.84)
2022	125,850 (11.16)	1398 (11.06)
2023	147,955 (13.13)	1757 (13.89)
2024	249,735 (22.15)	2457 (19.43)
2025	276,290 (24.51)	2875 (22.74)
Total	1,127,265 (100)	12,645 (100)

**Table 2:** Main characteristics of intoxication cases and individuals involved in opioid-notified poisonings.

Category	N (%)
Opioids implicated in opioid-notified poisoning notifications ^#^*	
Codeine	4887 (38.7%)
Tramadol	4297 (34.0%)
Morphine	2259 (17.9%)
Methadone	672 (5.3%)
Naltrexone	437 (3.5%)
Sex of individuals with reported intoxication	
Female	8891 (70.3)
Male	3752 (29.7)
Age of individuals with reported intoxication (years)	
0–25	4929 (39.3)
26–50	5992 (47.8)
51–75	1404 (11.2)
76–100	218 (1.7)
Missing data	106 (0.8)
Self-classified race/skin color category of individuals with reported intoxication	
White	7088 (56.0)
Brown	3892 (30.8)
Black	558 (4.4)
Yellow/Asian	109 (0.9)
Indigenous	19 (0.2)
Missing data	979 (7.7)
Education level of individuals with reported intoxication	
Illiterate	40 (0.3)
Incomplete elementary education	1164 (9.2)
Completed elementary education	880 (7.0)
Incomplete high school	1274 (10.1)
Completed high school	2832 (22.4)
Incomplete higher education	537 (4.2)
Completed higher education	931 (7.4)
Missing/Not applicable	4987 (39.4)
Geographic region of individuals with reported intoxication	
Southeast	5943 (47.0)
South	4116 (32.5)
Northeast	1364 (10.8)
Central-West	975 (7.7)
North	247 (2.0)
Employment status of individuals with reported intoxication	
Unemployed	1627 (12.9)
Formally employed	2072 (16.4)
Informally employed	1277 (10.1)
Employer	21 (0.2)
Retired	502 (4.0)
Missing/Ignored	7146 (56.5)
Place of intoxication	
Residence	10,823 (85.6)
Work-related settings	203 (1.6)
Institutional settings	395 (3.1)
External environment	190 (1.5)
Other/Missing	1034 (8.2)
Route of exposure *	
Digestive	11,674 (92.3)
Respiratory	261 (2.1)
Cutaneous	157 (1.2)
Parenteral	503 (4.0)
Ocular	11 (0.1)
Other/Missing	790 (6.2)
Circumstance of exposure	
Suicide attempt	9294 (73.5)
Abuse	542 (4.3)
Habitual use	428 (3.4)
Unintentional exposure	344 (2.7)
Other/Missing	2037 (16.1)
Type of exposure	
Acute–repeated	2338 (18.5)
Chronic	247 (2.0)
Acute–single	8045 (63.6)
Acute on chronic	196 (1.5)
Missing	1819 (14.4)
Type of care	
Hospital	9922 (78.4)
Outpatient	2413 (19.1)
Home care	76 (0.6)
None/Missing	234 (1.9)
Hospitalization	
No	5881 (46.5)
Yes	6195 (49.0)
Missing	569 (4.5)
Outcome	
Recovery without sequelae	9499 (75.1)
Recovery with sequelae	200 (1.6)
Death due to exogenous intoxication	202 (1.6)
Death due to another cause	152 (1.2)
Lost to follow-up	348 (2.8)
Missing	2244 (17.7)

^#^ The most frequently implicated opioids are shown in this table. * Percentages for opioid categories and exposure routes may exceed 100% because a single notification could include more than one reported opioid substance or route.

**Table 3:** Multivariable logistic regression models for selected outcomes among eligible opioid-notified poisoning cases.

Outcome	aOR	95% CI	*p*-Value
**Suicide attempt versus other eligible circumstances (N = 8464; AIC = 4335; R^2^McF = 0.101)**			
Age (years)	0.961	0.955–0.966	<0.001
Notification year	0.919	0.884–0.956	<0.001
Female versus male	1.980	1.687–2.340	<0.001
Residence versus external environment	8.946	6.289–12.724	<0.001
With co-exposure vs. without co-exposure	2.170	1.816–2.601	<0.001
**Hospitalization versus no hospitalization (N = 8237; AIC = 11376; R^2^McF = 0.0022)**			
Age (years)	1.001	1.000–1.001	0.083
Notification year	1.015	0.995–1.036	0.143
Female versus male	0.830	0.753–0.915	<0.001
Residence versus external environment	1.106	0.799–1.531	0.542
With co-exposure versus without co-exposure	1.135	1.016–1.268	0.025
**Other outcomes versus cure without sequelae (N = 8168; AIC = 7147; R^2^McF = 0.0088)**			
Age (years)	1.006	1.001–1.010	0.009
Notification year	1.053	1.024–1.084	<0.001
Female versus male	0.971	0.850–1.109	0.666
Residence versus external environment	0.397	0.277–0.569	<0.001
With co-exposure versus without co-exposure	1.107	0.947–1.294	0.201
Suicide attempt versus other eligible circumstances	0.749	0.610–0.921	0.006
**Serious outcomes versus cure without sequelae (N = 7124; AIC = 2221; R^2^McF = 0.0310)**			
Age (years)	1.017	1.009–1.026	<0.001
Notification year	1.045	0.985–1.108	0.141
Female versus male	0.959	0.730–1.260	0.766
Residence versus external environment	0.301	0.167–0.543	<0.001
With co-exposure versus without co-exposure	0.800	0.597–1.074	0.137
Suicide attempt versus other eligible circumstances	0.441	0.314–0.620	<0.001

aOR: adjusted odds ratio; CI: confidence interval. Logistic regression models were fitted using complete-case analysis. The model for other outcomes versus cure without sequelae included 8168 cases; the hospitalization model included 8237 cases; the suicide attempt model included 8464 cases; and the serious outcome model included 7124 cases. Odds ratios were re-expressed for selected variables by reversing the reference category to improve interpretability.

## Data Availability

The original contributions presented in the study are included in the article and Supplementary Material, further inquiries can be directed to the corresponding author. The data supporting the findings of this study are publicly available from the Departamento de Informática do Sistema Único de Saúde (DATASUS), based on records from the Sistema de Informações sobre Agravos de Notificação (SINAN). Data for 2019–2023 were accessed in March 2024, whereas data for 2024–2025 were accessed in February 2026. Because recent SINAN/DATASUS records may undergo late entry, correction, and updating, the most recent years should be interpreted as provisional.

## Supplementary Materials

The following supporting information can be downloaded at: https://www.mdpi.com/article/10.3390/ph19070994/s1, Figure S1: Observed and predicted incidence of opioid poisoning in Brazil by state. Table S1: Main characteristics of intoxication cases and individuals involved in heroin-notified poisonings; Table S2: Co-exposure patterns among the 9573 records with sufficient information for substance-level classification; Table S3: Bivariate analysis of factors associated with suicide attempt versus other eligible circumstances (confirmed intoxication, exposure, or adverse reaction) among eligible opioidrelated cases; Table S4: Suicide attempt versus other eligible circumstances according to official Brazilian self-classified race/skin color categories among eligible opioid-related poisoning cases; Table S5: Bivariate analysis of factors associated with hospitalization versus no hospitalization among eligible opioid-related cases (habitual use, abuse, or suicide attempts, with final classification as confirmed intoxication, exposure, or adverse reaction); Table S6: Hospitalization versus no hospitalization according to official Brazilian self-classified race/skin color categories among eligible opioid-related poisoning cases; Table S7: Bivariate analysis of factors associated with cure without sequelae versus other outcomes (cure with sequelae, death due to exogenous intoxication, death due to another cause, loss to follow-up, unknown) among eligible opioid-related cases; Table S8: Cure without sequelae versus other outcomes (cure with sequelae, death due to exogenous intoxication, death due to another cause, loss to follow-up, unknown) according to official Brazilian self-classified race/skin color categories among eligible opioid-related poisoning cases; Table S9: Bivariate analysis of factors associated with serious outcomes (recovery with sequelae, death due to exogenous intoxication, or death due to another cause) versus cure without sequelae among eligible opioid-related cases; Table S10: Serious outcomes versus cure without sequelae according to official Brazilian self-classified race/skin color categories among eligible opioid-related poisoning cases; Table S11: Comparison of the Poisson and negative binomial mixed models; Table S12: Coefficients of the mixed models for opioid poisonings in Brazil.
