# Supplementary material for: The Emerging Burden of Opioid Poisoning in Brazil, 2019–2025: A Nationwide Epidemiological and Toxicovigilance Analysis
Source: Pharmaceuticals (Basel). 2026 Jun 26;19(7):994. doi: 10.3390/ph19070994 (PMC13414491; doi:10.3390/ph19070994)
Supplement: Supplementary file 1 [file pharmaceuticals-19-00994-s001.zip › pharmaceuticals-4321958-supplementary.pdf]

SUPPLEMENTARY MATERIAL

The Emerging Burden of Opioid Poisoning in Brazil, 2019-2025: A Nationwide Epidemiological and Toxicovigilance Analysis

Luíza Siqueira Lima[a,b], Viviane Serra Melanda[c], Diancarlos Pereira de Andrade[a], Ana Tereza Bittencourt Guimarães[a,b], Cláudia Sirlene Oliveira[a,b]

[a] Instituto de Pesquisa Pelé Pequeno Príncipe, Curitiba, Brazil;

[b]Faculdades Pequeno Príncipe, Curitiba, Brazil;

[c]Secretaria de Estado da Saúde do Paraná - Curitiba (PR), Brazil.

*Corresponding author:

Oliveira, C.S. (claudia.sirlene@professor.fpp.edu.br)

S1. Heroin

Across the full compiled database, 105 notifications with heroin were examined (Supplementary Table 1). This corresponded to 0.8% of all 12,645 records. Notifications were concentrated in the most recent years, especially 2024 (n = 33) and 2025 (n = 20), although heroin-related mentions were present throughout the study period (2019: 10; 2020: 3; 2021: 12; 2022: 15; 2023: 12). Heroin-related cases occurred predominantly in men (79; 75.2%), while 26 cases (24.8%) involved women. Regarding self-classified race/skin color, White individuals were the most frequent group (48; 45.7%), followed by Brown (39; 37.1%) and Black (10; 9.5%). With respect to the circumstances of exposure, heroin was reported mainly in the context of abuse (71; 67.6%), followed by suicide attempt (22; 21.0%) and habitual use (8; 7.6%). In terms of final classification, most heroin-related notifications were recorded as confirmed intoxication (77; 73.3%), followed by exposure (16; 15.2%). Regarding outcome, 67 cases (63.8%) evolved to recovery without sequelae. However, heroin-related notifications also included 7 cases with recovery with sequelae (6.7%), 12 lost to follow-up (11.4%), 2 deaths due to exogenous intoxication (1.9%), and 1 death due to another cause (1.0%), in addition to ignored or missing outcomes. The general heroin profile was strongly marked by polysubstance exposure. The substances most frequently co-reported alongside heroin were cocaine (59 cases), alcohol (32), crack (21), K9 (21), fentanyl (20), and marijuana (14). This indicates that heroin-related notifications in this database were seldom isolated events and were more often embedded in broader patterns of mixed psychoactive substance use.

Supplementary Table S1. Main characteristics of intoxication cases and individuals involved in heroin-notified poisonings.

	Heroin-related notifications	N (%)
**Sex**		
	Female	26 (24.8)
	Male	79 (75.2)
**Regarding self-classified race/skin color**		
	White	48 (45.7)
	Brow	39 (37.1)
	Black	10 (9.5)
	Other/missing	8 (7.6)
**Circumstance of exposure**		
	Abuse	71 (67.6)
	Suicide attempt	22 (21.0)
	Habitual use	8 (7.6)
	Other/missing	4 (3.8)
**Final classification**		
	Confirmed intoxication	77 (73.3)
	Exposure	16 (15.2)
	Other/missing	12 (11.4)
**Outcome**		
	Recovery without sequelae	67 (63.8)
	Recovery with sequelae	7 (6.7)
	Lost to follow-up	12 (11.4)
	Death due to exogenous intoxication	2 (1.9)
	Death due to another cause	1 (1.0)
	Ignored outcome	9 (8.6)
	Missing outcome record	7 (6.7)
**Most frequent co-reported substances***		
	Cocaine	59 (56.2)
	Alcohol	32 (30.5)
	Crack	21 (20.0)
	K9	21 (20.0)
	Fentanyl	20 (19.0)
	Marijuana	14 (13.3)

* Co-reported substances were counted once per notification, even when repeated across multiple fields.

S2. Opioids-notified poisoning

Supplementary Table S2. Co-exposure patterns among the 9573 records with sufficient information for substance-level classification.

Co-exposure pattern	n (%)
Opioids and medications	8311 (86.76)
Multiple opioids and medications	459 (4.79)
Opioids, alcohol, and medications	190 (1.98)
Multiple opioids	160 (1.67)
Opioids and other illicit drugs	100 (1.04)
Opioids and alcohol	99 (1.03)
Opioids, medications, and drugs	67 (0.70)
Opioids, alcohol, and drugs	56 (0.58)
Multiple opioids and drugs	25 (0.26)
Opioids, alcohol, medications, and drugs	15 (0.16)
Opioids, medications, and caffeine	9 (0.09)
Opioid, medications and rodenticide	9 (0.09)
Multiple opioids, alcohol, and medications	8 (0.08)
Multiple opioids and alcohol	6 (0.06)
Opioid, medications and hypochlorite	6 (0.06)
Multiple opioids, medications, and drugs	6 (0.06)
Opioid and caffeine	5 (0.05)
Multiple opioids, alcohol, and drugs	5 (0.05)
Opioid and hypochlorite	4 (0.04)
Opioid, medications, and K-othrine®	4 (0.04)
Opioid, medications and chumbinho*	3 (0.03)
Opioid and rodenticide	2 (0.02)
Opioid, medications and insecticide	2 (0.02)
Opioid, medications, poison	2 (0.02)
Multiple opioids and chumbinho*	2 (0.02)

Opioid, alcohol, and hypochlorite	2 (0.02)
Opioid and chumbinho	1 (0.01)
Opioid, insecticide	1 (0.01)
Opioid and multi-purpose cleaner	1 (0.01)
Opioid and perfume	1 (0.01)
Opioid and hydrogen peroxide	1 (0.01)
Opioid, rodenticide, and cockroach poison	1 (0.01)
Opioid, alcohol and gasoline	1 (0.01)
Opioid, alcohol, medications, and chumbinho*	1 (0.01)
Opioid, alcohol, medications, and hypochlorite	1 (0.01)
Opioid, alcohol, and rodenticide	1 (0.01)
Opioid, medications, and glass powder	1 (0.01)
Opioid, medications and soap	1 (0.01)
Multiple opioids, medications, drugs, and hypochlorite	1 (0.01)
Multiple opioids, medications, and hypochlorite	1 (0.01)
Multiple opioids, medications, and rodenticide	1 (0.01)
Multiple opioids and rodenticide	1 (0.01)

*Illegal rat poison, unregistered rodenticide

Supplementary Table S3. Bivariate analysis of factors associated with suicide attempt versus other eligible circumstances (confirmed intoxication, exposure, or adverse reaction) among eligible opioid-related cases.

	Category	Other circumstances	Suicide attempt	p-value
**Sex**	Female	465 (54.5)	6268 (73.1)	
	Male	388 (45.5)	2304 (26.9)	<0.001
**Employment status**	Other	371 (73.5)	3699 (75.4)	
	Unemployed	134 (26.5)	1208 (24.6)	0.342
**Place of exposure**	External environment	63 (9.0)	94 (1.2)	
	Residence	634 (91.0)	7713 (98.8)	<0.001
**Co-exposure**	With co-exposure	602 (70.6)	7056 (82.3)	
	Without co-exposure	251 (29.4)	1517 (17.7)	<0.001
**Hospitalization**	Hospitalized	430 (51.7)	4423 (53.1)	
	Not hospitalized	402 (48.3)	3905 (46.9)	0.432
**Outcome**	Cure without sequelae	604 (74.9)	6929 (83.5)	<0.001
	Other outcomes	202 (25.1)	1366 (16.5)	

[a]Pearson's chi-square test

Supplementary Table S4. Suicide attempt versus other eligible circumstances according to official Brazilian self-classified race/skin color categories among eligible opioid-related poisoning cases.

**Official Brazilian self-classified race/skin color category**	**Other eligible circumstances n (%)**	**Suicide attempt n (%)**	**Total**
Yellow/Asian	7 (8.9)	72 (91.1)	79
White	481 (8.8)	5000 (91.2)	5481
Indigenous	2 (15.4)	11 (84.6)	13
Brown	261 (9.3)	2537 (90.7)	2798
Black	34 (7.9)	395 (92.1)	429
Ignored	61 (10.3)	530 (89.7)	591

Pearson's $\chi^2 = 3.21$; df = 5; p = 0.667. Fisher's exact test p = 0.575.

Supplementary Table S5. Bivariate analysis of factors associated with hospitalization versus no hospitalization among eligible opioid-related cases (habitual use, abuse, or suicide attempts, with final classification as confirmed intoxication, exposure, or adverse reaction).

	Category	Hospitalized	No hospitalized	p-value
**Sex**	Female	3395 (70.0)	3137 (72.9)	0.002
	Male	1458 (30.0)	1169 (27.1)	
**Employment status**	Other	2082 (75.9)	1907 (74.4)	0.225
	Unemployed	662 (24.1)	655 (25.6)	
**Place of exposure**	External environment	79 (1.8)	72 (1.9)	0.851
	Residence	4312 (98.2)	3810 (98.1)	
**Co-exposure**	With co-exposure	3979 (82.0)	3456 (80.2)	0.033
	Without co-exposure	874 (18.0)	851 (19.8)	
**Outcome**	Cure without sequelae	3618 (77.7)	3757 (89.3)	<0.001
	Other outcomes	1039 (22.3)	451 (10.7)	
**Circumstance of exposure**	Other eligible circumstances	430 (8.9)	402 (9.3)	0.432
	Suicide attempt	4423 (91.1)	3905 (90.7)	

[a]Pearson's chi-square test

*Table 5 includes only eligible cases with non-missing hospitalization status with the predefined criteria for this analysis; therefore, its denominator differs from the full descriptive dataset presented in Table 2.

Supplementary Table S6. Hospitalization versus no hospitalization according to official Brazilian self-classified race/skin color categories among eligible opioid-related poisoning cases.

Official Brazilian self-classified race/skin color category	Hospitalized n (%)	Not hospitalized n (%)	Total
Yellow/Asian	37 (50.0)	37 (50.0)	74
White	2863 (53.4)	2495 (46.6)	5358
Indigenous	5 (38.5)	8 (61.5)	13
Brown	1421 (52.4)	1291 (47.6)	2712
Black	185 (45.3)	223 (54.7)	408
Ignored	325 (57.9)	236 (42.1)	561

Pearson's $\chi^2 = 17.3$; df = 5; p = 0.004.

Supplementary Table S7. Bivariate analysis of factors associated with cure without sequelae versus other outcomes (cure with sequelae, death due to exogenous intoxication, death due to another cause, loss to follow-up, unknown) among eligible opioid-related cases.

	Cure without sequelae n (%)	Other outcomes n (%)	p-value[a]
**Sex**			
Female	5432 (72.1)	1085 (69.2)	0.024
Male	2101 (27.9)	482 (30.8)	
**Employment status**			
Unemployed	1125 (25.1)	186 (24.3)	0.673
Other	3364 (74.9)	578 (75.7)	
**Place of exposure**			
External environment	95 (1.4)	49 (3.7)	<0.001
Residence	6791 (98.6)	1270 (96.3)	
**Co-exposure**			
With co-exposure	6111 (81.1)	1280 (81.6)	0.638
Without co-exposure	1422 (18.9)	288 (18.4)	

[a]Pearson's chi-square test

Supplementary Table S8. Cure without sequelae versus other outcomes (cure with sequelae, death due to exogenous intoxication, death due to another cause, loss to follow-up, unknown) according to official Brazilian self-classified race/skin color categories among eligible opioid-related poisoning cases.

Official Brazilian self-classified race/skin color category	Cure without sequelae n (%)	Other outcomes n (%)	Total
Yellow/Asian	61 (80.3)	15 (19.7)	76
White	4484 (84.3)	836 (15.7)	5320
Indigenous	9 (75.0)	3 (25.0)	12
Brown	2233 (82.9)	461 (17.1)	2694
Black	336 (81.6)	76 (18.4)	412
Ignored	389 (69.5)	171 (30.5)	560

Pearson's $\chi^2 = 79.4$; df = 5; $p < 0.001$.

Supplementary Table S9. Bivariate analysis of factors associated with serious outcomes (recovery with sequelae, death due to exogenous intoxication, or death due to another cause) versus cure without sequelae among eligible opioid-related cases.

	Category	Cure without sequelae	Serious outcomes	p-value
**Sex**	Female	5432 (72.1)	225 (65.6)	0.009
	Male	2101 (27.9)	118 (34.4)	
**Employment status**	Other	3364 (74.9)	170 (78.0)	0.310
	Unemployed	1125 (25.1)	48 (22.0)	
**Place of exposure**	External environment	95 (1.4)	15 (5.6)	<0.001
	Residence	6791 (98.6)	253 (94.4)	
**Co-exposure**	With co-exposure	6111 (81.1)	254 (74.1)	
	Without co-exposure	1422 (18.9)	89 (25.9)	0.001
**Hospitalization**	Hospitalized	3618 (49.1)	222 (65.9)	<0.001
	Not hospitalized	3757 (50.9)	115 (34.1)	
**Circumstance**	Other circumstances	604 (8.0)	80 (23.3)	<0.001
	Suicide attempt	6929 (92.0)	263 (76.7)	

[a]Pearson's chi-square test

Supplementary Table S10. Serious outcomes versus cure without sequelae according to official Brazilian self-classified race/skin color categories among eligible opioid-related poisoning cases.

Official Brazilian self-classified race/skin color category	Cure without sequelae n (%)	Serious outcomes n (%)	Total
Yellow/Asian	61 (98.4)	1 (1.6)	62
White	4484 (95.6)	206 (4.4)	4,690
Indigenous	9 (90.0)	1 (10.0)	10
Brown/Parda	2233 (95.7)	101 (4.3)	2,334
Black	336 (95.7)	15 (4.3)	351
Ignored	389 (95.6)	18 (4.4)	407

Pearson's $\chi^2 = 1.91$; df = 5; p = 0.861. Fisher's exact test p = 0.777.

S3. Predictive Models

The negative binomial model showed a better fit (lower AIC/BIC and higher LogLik), demonstrating overdispersion in the data (phi = 8.04) and inadequacy of the Poisson model (Supplementary Table 11). The effect of year was positive and significant in both models ($p < 0.001$), indicating increasing incidence over time. In the negative binomial model, the incidence rate increased by 15.6% per year (IRR = 1.156) (Supplementary Table 12).

Supplementary Table S11. Comparison of the Poisson and negative binomial mixed models.

Model	AIC	BIC	LogLik	Dispersion (phi)
Poisson	1802.5	1812.0	-898.3	—
Negative binomial	1265.8	278.4	-628.9	8.04

Supplementary Table S12. Coefficients of the mixed models for opioid poisonings in Brazil.

Model	Parâmetro	Estimate (β)	SE	z	p-value	IRR (exp β)
Poisson	Intercept	-13.028	0,173	-75,18	<0,001	—
	Year	0.110	0.006	18.34	<0.001	1.116
Negative binomial	Intercept	-13.143	0.182	-72.39	<0.001	—
	Year	0.145	0.016	9.00	<0.001	1.156

Supplementary Figure S1. Observed and predicted incidence of opioid poisoning in Brazil by state

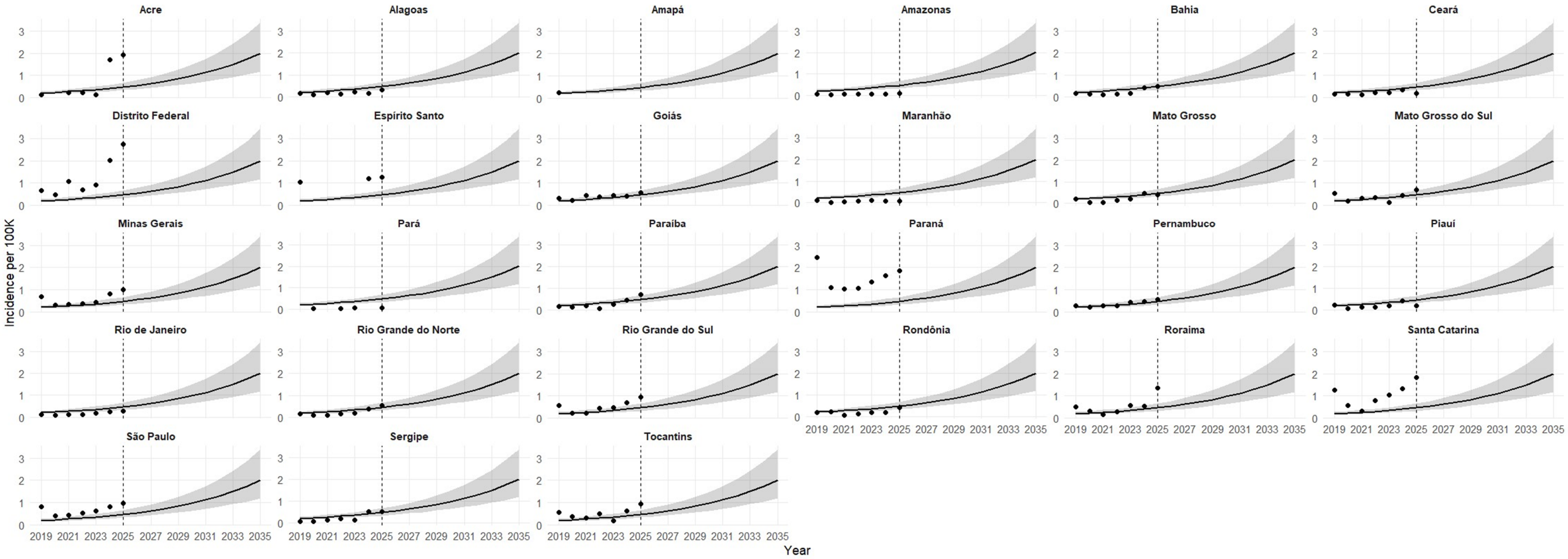

Note: Negative binomial mixed model with 95% CI. An increasing trend in opioid poisoning incidence across Brazilian federative units is observed over the analyzed period. The points represent the observed values between 2019 and 2025, while the solid line indicates the incidence estimated by the negative binomial mixed model. The shaded area corresponds to the 95% confidence interval of the estimates. The vertical dashed line marks the beginning of the projection period (after 2025), during which a progressive increase in incidence is observed, accompanied by widening confidence intervals, reflecting greater uncertainty in future estimates.
